# Flux sampling is a powerful tool to study metabolism under changing environmental conditions

**DOI:** 10.1038/s41540-019-0109-0

**Published:** 2019-09-02

**Authors:** Helena A. Herrmann, Beth C. Dyson, Lucy Vass, Giles N. Johnson, Jean-Marc Schwartz

**Affiliations:** 1 Department of Earth and Environmental Sciences, University of Manchester, Manchester, UK; 2 School of Biological Sciences, University of Manchester, Manchester, UK; 3 Present Address: Department of Animal and Plant Sciences, University of Sheffield, Sheffield, UK; 4 Present Address: Bristol Veterinary School and Department of Population Health Sciences, University of Bristol, Bristol, UK

**Keywords:** Biochemical networks, Plant sciences

## Abstract

The development of high-throughput ‘omic techniques has sparked a rising interest in genome-scale metabolic models, with applications ranging from disease diagnostics to crop adaptation. Efficient and accurate methods are required to analyze large metabolic networks. Flux sampling can be used to explore the feasible flux solutions in metabolic networks by generating probability distributions of steady-state reaction fluxes. Unlike other methods, flux sampling can be used without assuming a particular cellular objective. We have undertaken a rigorous comparison of several sampling algorithms and concluded that the coordinate hit-and-run with rounding (CHRR) algorithm is the most efficient based on both run-time and multiple convergence diagnostics. We demonstrate the power of CHRR by using it to study the metabolic changes that underlie photosynthetic acclimation to cold of *Arabidopsis thaliana* plant leaves. In combination with experimental measurements, we show how the regulated interplay between diurnal starch and organic acid accumulation defines the plant acclimation process. We confirm fumarate accumulation as a requirement for cold acclimation and further predict *γ*–aminobutyric acid to have a key role in metabolic signaling under cold conditions. These results demonstrate how flux sampling can be used to analyze the feasible flux solutions across changing environmental conditions, whereas eliminating the need to make assumptions which introduce observer bias.

## Metabolic modeling: flux sampling to study acclimation

Flux sampling minimizes observer bias and is therefore a powerful tool for studying metabolic acclimation. Unlike other methods, flux sampling can be used without assuming that metabolism is optimized towards a single objective. This is particularly useful when studying metabolism in changing environments. A team lead by Jean-Marc Schwartz compared three different flux sampling algorithms in order to identify the most appropriate method to analyze changes in metabolic phenotypes. They applied flux sampling to several large-scale networks of plant metabolism to predict metabolic properties across changing temperatures. In order to breed plants for global climate changes, the limits on photosynthesis and metabolism across different environmental conditions need to be understood. Here, they used flux sampling methods to understand changes in photosynthesis and carbon accumulation in cold temperatures. They showed how an intricate balance between carbon and nitrogen metabolism is crucial for acclimation to cold.

## Introduction

High-throughput technologies have resulted in a rapid increase in available ‘omic data sets.^1^ Large-scale metabolic networks constructed using these data integrate known and predicted metabolic pathways.^2^ These large-scale networks can be constrained using experimental data and the system behavior can be analyzed using metabolic modeling. Metabolism describes a cellular phenotype under given conditions, and changes in metabolite concentrations and reaction fluxes can be used to assess a cellular response to changing environmental conditions.

With the existence of large-scale metabolic networks comes the need to have appropriate modeling techniques available for their analysis. The majority of techniques for analyzing large-scale metabolic networks fall within the paradigm of constraint-based modeling (CBM).^3^ CBM imposes stoichiometric constraints on the metabolic reactions and analyzes the possible flux solutions at steady state. Because the system is assumed to be at steady state, even genome-scale models can be solved at little computational expense.

Two of the most widely used forms of CBM are flux balance analysis (FBA) and flux variability analysis (FVA).^4^ The key feature of FBA and FVA is that they compute the steady state of a model using an objective function. The objective function defines a reaction that is to be maximized or minimized when solving the system under the set constraints. A typical objective is “maximum biomass production”, whereby essential macromolecules such as proteins and lipids are defined at known ratios or quantities in an outgoing reaction of the metabolic system.^5,6^ FBA computes single steady-state solutions, which satisfy the objective. However, often multiple solutions exist and their range can be computed using FVA.^7^ FVA provides no indication as to whether all single-point solutions within the range are feasible and which solutions are the most likely. In order to reduce the number of feasible solutions of FBA and to further constrain the feasible flux ranges returned by FVA, it is common practice to introduce multiple objective functions.^8^

However, defining one or multiple objective function(s) intrinsically introduces an observer bias as to what the main “goal” of the cell is, in the context of the analysis.^9^ Although biomass production as estimated by FBA was seen to match experimental data in *Escherichia coli*,^10^ this may not be an appropriate objective when studying short-term environmental changes. In the green algae *Chlamydomonas reinhardtii*, a ^13^C-metabolic flux analysis was shown to contradict the assumptions of a prior FBA analysis by demonstrating that maximum biomass and maximum ATP production cannot stand alone as cellular objectives.^11^ Optimal growth conditions, to which most objective functions are tailored, are an exception in natural environments.^5^ Evolutionarily, metabolism is most likely optimized for overall robustness across many conditions, rather than a single condition-specific objective. *Bacillus subtilis* mutants, which outperform the wild-type in terms of biomass production in control conditions have been found experimentally; the wild-type, however, is more robust to both environmental and genetic perturbations and therefore holds an evolutionary advantage.^12^

Understanding the optimization process that allows an organism to tolerate changing environmental conditions is of particular interest in crop sciences, where increasing yields will need to be achieved, despite rapidly changing environmental conditions. Owing to their sessile nature, plants are exposed to frequent and sometimes extreme environmental fluctuations. Plant metabolism can therefore be presumed to hold an inherent robustness to changing environmental conditions. Furthermore, plant metabolism is highly intricate as it includes multi-cellular autotrophic and heterotrophic tissues with complex cellular compartmentalization. Analyses of plant metabolism and of plant metabolic strategies can therefore be considered a comprehensive case study of metabolism in general. Genetic modifications do not always produce a desired effect owing to network robustness:^13,14^ in crop sciences, optimization for increased yield in control conditions rather than in changing environments is a likely explanation for discrepancies between laboratory and in field results.^15,16^

If we wish to use CBM techniques to assess network robustness and phenotypic plasticity, we must be able to capture all alternative solutions and the probability with which they occur (the solutions space) of a metabolic network across different conditions. To calculate the exact properties of the solution space, we can use mathematical techniques such as convex analysis and vertex enumeration;^17^ however, owing to their computational intensiveness, these methods are only efficient when applied to small, simple networks^18^ or genome-scale models with one or more objective function.^19^ When wanting to analyze genome-scale metabolic networks without the use of an objective function, such approaches cannot practically be applied. Sampling of feasible flux solutions provides a realistic alternative.

Flux sampling generates a sequence of feasible solutions (called a chain) that satisfy the network constraints, until the entire solution space is analyzed. Enough samples need to be generated for the samples to provide an accurate representation of the feasible solution space.^18^ A chain of samples is said to have converged once it contains enough samples to give an accurate representation of the solution space.^20^ Flux sampling provides information both on the range of feasible flux solutions (similar to FVA) but also on their probability. Importantly, unlike FBA, flux sampling does not require (but also does not exclude the option for) an objective function to be specified. Therefore, flux sampling methods hold great potential for analyzing optimization strategies that are not defined by clear objectives such as a simple biomass reactions.

When plants are exposed to a change in environmental conditions, such as temperature, which last only for a few days, optimizing biomass production during this period is arguably of secondary importance; sustaining metabolic function with minimal cost may be a higher priority to plants. For example, the allocation of carbon into different transient storage compounds accumulated to maintain cellular processes, has been shown to change when plants are exposed to environmental stresses.^21–25^ Starch, malate, and fumarate are the three major carbon storage compounds which accumulate during the day in leaves of the model plant *Arabidopsis thaliana*.^21,26,27^ Increased cytosolic fumarate accumulation is a known cold response of *A*. *thaliana* leaves and has been linked to an increased photosynthetic capacity that sustains metabolism in cooler temperatures.^24^ Evidently, tight regulation of carbon partitioning is required for successful cold acclimation. Given the large number of reactions and pathways involved in linking primary carbon assimilation to its downstream storage products starch, malate, and fumarate, CBM seems appropriate. Of the CBM methods available, flux sampling allows us to gain a detailed understanding of the solution space and the interdependence of the different carbon stores under different temperature conditions, without imposing the constraint of an objective function.

Multiple large-scale metabolic networks of the model plant *A*. *thaliana* have been constructed.^28–32^ Here, we used three of them to formally assess for the efficiency of existing and easily accessible flux sampling algorithms: the coordinate hit-and-run with rounding (CHRR),^33^ the artificially centered hit-and-run (ACHR)^34^ and the optimized general parallel (OPTGP)^35^ algorithms. We identified the most efficient sampling method based on run-time and convergence, and applied it to study plant acclimation to cold. We experimentally measured diurnal CO2 uptake and organic carbon accumulation of *A*. *thaliana* in control and cold conditions. By constraining a leaf metabolic model to the two conditions and using an appropriate flux sampling algorithm, we were able to explore inherent metabolic robustness to temperature and predict the metabolic changes required to support a photosynthetic acclimation response to cold.

Although flux sampling has previously been applied as a technique for studying the solution space of metabolic networks,^29,36,37^ this will, to our knowledge, be the first time that the available algorithms are formally compared with one another in the context of metabolic modeling, and that flux sampling is applied to study network robustness across changing environmental conditions.

## Results

### Both run-time and convergence are fastest when using CHRR in MATLAB

We compared the run-time and convergence of the CHRR, ACHR, and OPTGP algorithms using three metabolic models of *A*. *thaliana*. We tested the run-times of 500,000, 5,000,000, and 50,000,000 samples (*S*), of which 5000 were stored and the rest were discarded with constant measures of thinning. For *S* = 50,000,000, the CHRR algorithm was 2.5 times faster than the OPTGP and 5.3 times faster than the ACHR for the Arnold model (Fig. 2). This difference in speed increases with model complexity, such that, for the Poolman model, the CHRR was 3.3 times faster than the OPTGP and 8.0 times faster than the ACHR (Fig. 2). The OPTGP was run in two parallel processes; however, even when running it as a single process it is faster than the ACHR. Although we cannot exclude the fact that MATLAB may have a faster connection to Gurobi than Python, flux sampling is fastest when using the CHRR setup as available in the COBRA toolbox for MATLAB.

**Fig. 1:**
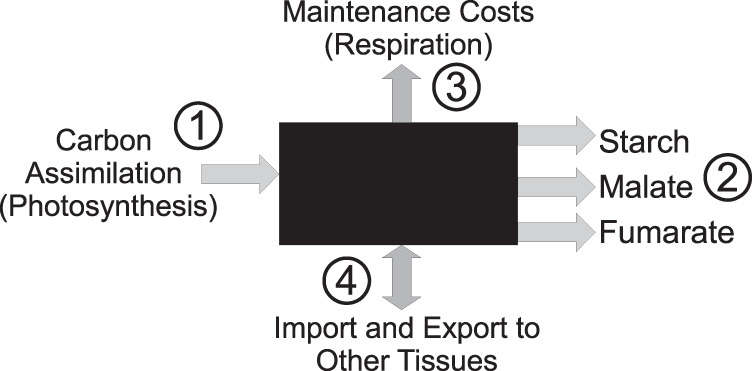
Summary of the general influx and outflux of the Arnold model, as set up in our analysis. Differences in pathways linking carbon assimilation and diurnal carbon storage were compared across the two temperatures (20 °C and 4 °C) in order to predict metabolic changes required for cold acclimation of *A. thaliana*

**Fig. 2:**
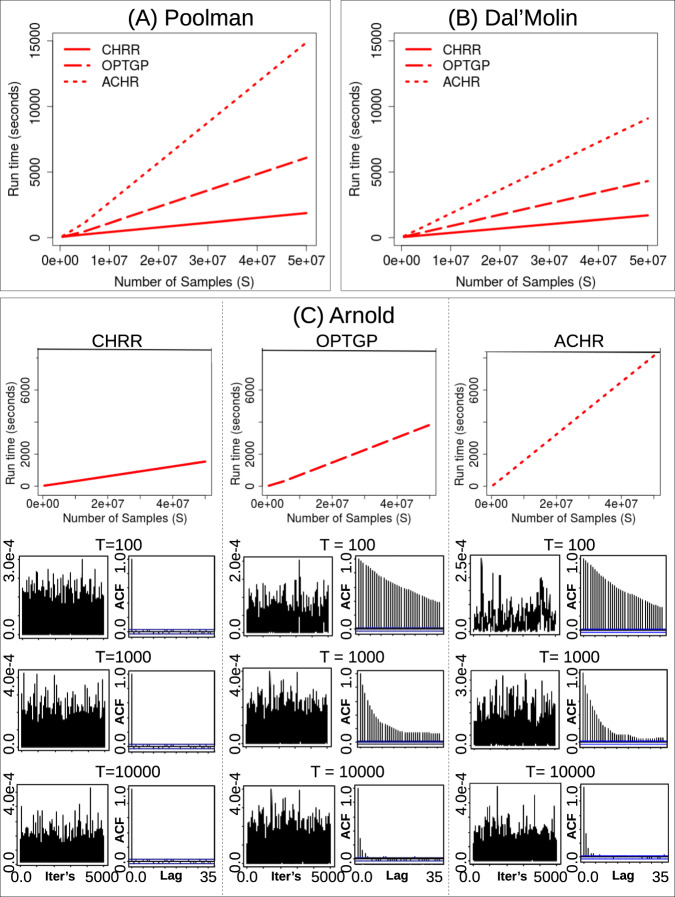
Run-times for each of the algorithms on their respective platforms (CHRR: MATLAB; ACHR: Python; OPTGP: Python) when sampling flux solution for the Poolman **a**, Dal’Molin **b**, and Arnold model **c**. Trace and autocorrelation (ACF) plots (showing sampling chains and sample dependence, respectively) for chains of length 5000 with thinnings of *T* = 100, 1000, and 10,000 of the biomass reaction, as obtained when using each of the algorithms on the Arnold model

The number of reactions that did not satisfied the convergence criteria were assessed for each chain (Table 1). The longer run-times of ACHR and OPTGP implementations in Python are not outweighed by faster convergence. In fact, as the three pilot chains with varying thinnings show, the CHRR algorithm, across all model reactions, converges the fastest, with the lowest number of samples required for convergence, the least amount of autocorrelation, and the lowest discrepancy between chains (Table 1). We confirmed this difference in convergence by inspecting trace and auto-correlations plots of individual reactions, such as those for the biomass reaction of the Arnold model shown in Fig. 2 (C). CHRR shows little dependence between consecutive samples even with a thinning of *T* = 100, whereas OPTGP and ACHR show low levels of autocorrelation only when *T* = 10,000.

**Table 1:** Convergence diagnostics comparing three chains of 5000 samples run for all reactions in the Poolman, Arnold, and Dal’Molin models using the ACHR, OPTGP, and CHRR algorithm with the indicated thinning

Model	Sampler	Thinning	Convergence diagnostics			
			*N*_max_ (Raftery & Lewis)	% *I* > 5 (Raftery & Lewis)	% *Z* > 1.28 (Gweke)	% IPSRF < 0.9 or IPSRF > 1.1
Poolman	ACHR	100	2862482	53	28	30
		1000	1557990	46	23	16
		10,000	1083360	41	20	14
	OPTGP	100	1664187	53	22	13
		1000	1099330	45	21	11
		10,000	1717480	40	17	6
	CHRR	100	303880	15	14	0
		1000	27906	0	12	0
		10,000	4636	0	10	0
Arnold	ACHR	100	385177	49	39	9
		1000	312384	12	34	3
		10,000	323570	9	23	0
	OPTGP	100	910210	45	36	7
		1000	1538190	12	30	3
		10,000	1679346	9	32	17
	CHRR	100	122760	17	24	0
		1000	17508	0	21	0
		10,000	4198	0	19	0
Dal’Molin	ACHR	100	41951912	37	13	17
		1000	4982459	28	12	10
		10,000	1544792	23	10	6
	OPTGP	100	1834104	37	17	14
		1000	1891665	27	11	6
		10,000	1700907	22	11	3
	CHRR	100	550776	10	16	3
		1000	387456	4	16	3
		10,000	226856	3	13	2

Three samples sets (chains) were computed for each reaction using the indicated models, algorithms and thinnings. *N*_max_ indicates the maximum number of total samples suggested by the Raftery & Lewis diagnostic. % *I* > 5 and % *z* > 1.28 indicate the average percentage of reactions which do not pass the Raftery & Lewis and Gweke convergence diagnostics according to the three chains. The three chains were used for each reaction in order to compare within and between sample differences as indicated by the interval-based potential scale reducing factor (IPSRF) diagnostic. The percentage of reactions with IPSRF < 0.9, IPSRF > 1.1 is shown

Our results show differences in the outcomes when convergence is reached according to the different convergence diagnostics. All convergence diagnostics agree that the CHRR performs best (Table 1). According to the Raftery & Lewis and the IPSRF diagnostics, all of the flux samples of reactions in the Arnold and Poolman models converge in < 5000 samples with a thinning constant of 10,000 when using CHRR. The fact that such large numbers of samples are required for model convergence shows that, owing to the irregular solution shape of genome-scale metabolic networks, autocorrelation in chains is a common problem and must be overcome and tested for using appropriate convergence diagnostics. Analyzing samples that have not achieved convergence can lead to incorrect conclusions about the metabolic fluxes under study. Currently, many applications of the ACHR, OPTGP, and CHRR algorithms to biological networks do not report whether convergence has actually been achieved.^29,37–39^

Previous comparisons between the ACHR and CHRR algorithms^33^ have been made using 15 different metabolic models but were based only on a single convergence diagnostic, the potential scale reduction factor (PSRF).^40^ Notably, different convergence diagnostics test different features and may not always be in good agreement. Therefore, more than one diagnostic should be used to confirm that the sampling chain is likely to have reached convergence.^20,41^ The ACHR and OPTGP algorithms have been compared using five genome-scale metabolic models and three different convergence diagnostics, including the PSRF.^35^ However, the PSRF assumes a normal distribution of solutions,^42^ which is questionable given that sampling is most needed when the distribution of flux estimates is non-normal,^41^ as is often the case in metabolic modeling.

### CHRR-based flux sampling generates verifiable hypotheses concerning plant acclimation to cold

**Fig. 3:**
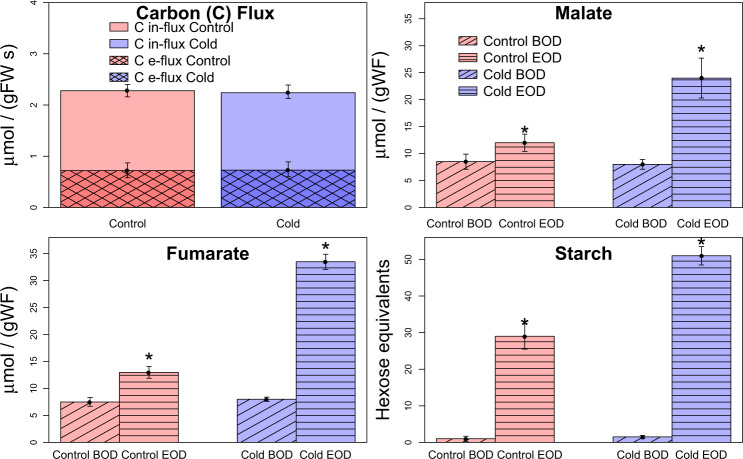
Carbon influx and outflux and organic carbon compound accumulation in leaves. Measurements were taken at control conditions (red) and after 7 days of cold treatment (blue), at the beginning of day (BOD) and at the end of day (EOD), using infrared gas analysis and enzyme assays.^24^ The s.e.m. of the 3–4 replicates for each measurement is shown as error bars. The above data (excluding carbon outflux) were converted to $\frac{{mmol}}{{gFW \cdot Day}}$ and used to constrain the Arnold model as outlined in the method. Significant differences between measurements across the two temperature conditions are indicated by an asterisk

When plants of *A*. *thaliana* are transferred from 20 °C to 4 °C, photosynthesis is inhibited.^24^ Metabolism slows down in cooler temperatures and, in order to sustain normal metabolic functions, plants need to acclimate their CO_2_ uptake, altering the concentrations of metabolic enzymes to achieve a new steady state.^24,43,44^ After 7 days of cold, we observe that the *A*. *thaliana* wild-type Col-0 is able to achieve the same level of photosynthesis as measured in control conditions (Fig. 3). The allocation of carbon to the three main carbon storage compounds, malate, fumarate, and starch, shifts in the cold as part of the metabolic acclimation response. After 7 days of acclimation both photosynthesis and transient carbon accumulation attain a new cold acclimated state. Most notably, after 7 days of cold treatment a larger proportion of carbon is partitioned into fumarate (Fig. 3).

**Fig. 4:**
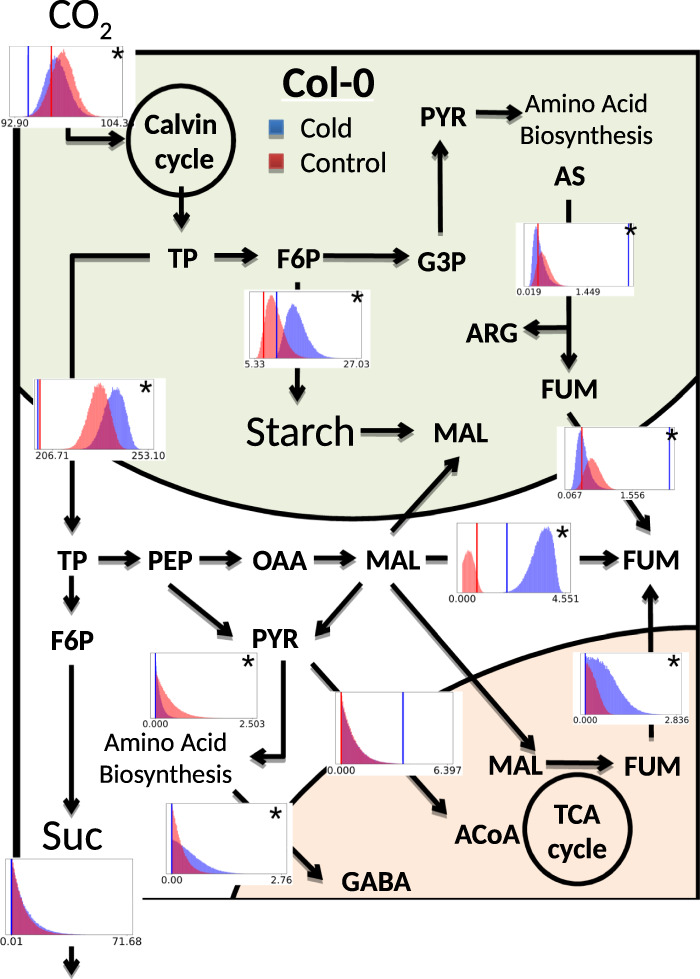
Flux sampling distributions of key reactions linking photosynthetic input (CO_2_) to transient carbon storage for control (red) and cold (blue) conditions. Condition-specific carbon assimilation and fumarate (FUM), malate (MAL), and starch accumulation were constrained according to experimentally measured results. Resulting fluxes of reactions including triose phosphate (TP), fructose 1,6-bisphosphate (F6P), glyceraldehyde 3-phosphate (G3P), pyruvate (PYR), arginosuccinate (AS), arginine (ARG), phosphoenolpyruvate carboxylase (PEP), oxaloacetate (OAA), *γ*-aminobutyric acid (GABA), acetyl coenzyme A (ACoA), and sucrose (Suc) are shown. We overlaid FBA results for maximum biomass production (under the same model constraints as applied for the sampling) as vertical blue and red bars over the flux sampling distributions. Reactions for which the two distributions are significantly different (*p* < 0.001; Kruskal–Wallis) are marked with an asterisk in the top right corner

Using the experimental data shown in Fig. 3 to constrain the CO_2_ input and the malate, fumarate, and starch accumulation reactions using the Arnold model (please see methods for further details), we were able to compute converged flux sampling distributions for all reactions. We did so for both control and cold conditions, which allowed us to overlay the sampling distributions of reaction fluxes and to assess changes required in plant metabolic behavior for acclimation (Fig. 4).

In order to demonstrate how the application of an objective function for an FBA analysis can lead to vastly different conclusions, we have overlaid FBA results for maximum biomass production (under the same model constraints as applied for the sampling) over the flux sampling distributions (Fig. 4). This further emphasizes how an objective function, if inappropriate for the analyses under consideration, can be misleading.

Both sucrose export to other tissues and cytosolic pyruvate production as a precursor for the tricarboxylic acid (TCA) cycle are predicted to be unchanged. The model suggests that, given equal carbon assimilation in control and cold conditions, cellular maintenance and export functions are supported equally in both conditions on the time scale considered. Although sucrose export is difficult to measure experimentally, we observed the rate of respiration in the cold to be the same as in control conditions (Fig. 3). Given that, for both conditions, the model shows equal fluxes from cytosolic pyruvate into the TCA cycle (Fig. 4), which feeds directly into respiration, this model prediction is in agreement with our experimental data.

For fumarate (and other metabolites not shown) the model predicts a shift in cellular compartmentalization with temperature. Model results show an increase in fumarate export from the mitochondrion into the cytosol in the cold. The reverse is shown for fumarate export from the chloroplast, where it is produced via the breakdown of arginosuccinate (Fig. 4). Leaves developed in the cold have increased cytoplasmic and decreased vacuolar volumes;^45^ a reshuffling of metabolites across cellular compartments has therefore previously been proposed as an important temperature acclimation response.^46^

Model results suggest an increase in flux from cytosolic malate to fumarate via cytosolic fumarase (FUM2) (Fig. 4). This is consistent with previous experimental results from mutant studies that show that the *fum2*.*2* mutant of Col-0 is unable to acclimate to cold.^24^ This reaction is thus essential for photosynthetic acclimation to cold.

### Flux sampling distributions suggest a link between carbon and nitrogen metabolism to support cold acclimation

The sampling distributions suggest a trade off between increased carbon compound accumulation and decreased amino-acid production (Fig. 4), linking nitrogen and carbon metabolism. Synthesis of *γ*-aminobutyric acid (GABA), however, is predicted to increase in the cold. GABA has previously been reported to accumulate in response to environmental stresses, including cold treatment.^47^ GABA has been suggested as a signaling molecule of the carbon to nitrogen status in plant leaves and evidence for its role in regulating nitrate uptake exists for both rapeseed and *A*. *thaliana*.^48–50^ *A*. *thaliana* plants in the cold show increased nitrogen assimilation compared with those in control conditions.^51^

If GABA is indeed involved in carbon:nitrogen signaling,^48^ it may be counteracted by the increased accumulation of malate. Increased levels of malate have previously been shown to suppress nitrate reductase expression and activity in tobacco leaves.^52^ Malate levels in Col-0 may be kept below a certain threshold, by redirecting carbon to fumarate, thereby supporting adequate nitrogen assimilation and an increased photosynthetic capacity. This hypothesis is supported by the observation that *A*. *thaliana* mutants, which show increased levels of malate and decreased levels of fumarate, grow significantly less well in high-nitrogen conditions than Col-0.^22^ Fumarate has few known metabolic functions in *A*. *thaliana* leaves,^26^ and may thus serve the purpose of a carbon storage buffer in changing environmental conditions.

## Discussion

Based on run-time and platform, CHRR is faster than OPTGP, which is itself faster than ACHR. CHRR also converges faster than both OPTGP and ACHR. Users with unrestricted access to MATLAB are therefore recommended to use CHRR. For those who wish to work using an open-source platform, OPTGP is recommended over ACHR; in general, OPTGP converges faster than ACHR, has a shorter run-time and allows for parallel processes.

When running sampling algorithms, sets of flux samples are produced for each reaction in the model. Here, we have tested convergence for all reactions of the models using three different sample chains. We have highlighted the importance of checking for convergence using different diagnostics when analyzing an irregular solution space of large networks. If only a subset of reaction fluxes are of interest, only those distributions will have to be checked for convergence. Flux sampling provides a powerful tool for exploratory analyses assessing metabolic differences across different environmental conditions. Our results further confirm the notion that it is not possible to fully automate convergence analyses using a single diagnostic^20^ and that results should confirmed via manual inspections of trace and autocorrelation plots.

Flux sampling is currently an under-utilized technique in the metabolic modeling of large-scale networks. Using cold acclimation of the model plant *A*. *thaliana*, we demonstrate how flux sampling can be used effectively to analyze alternative feasible solutions across multiple conditions whilst eliminating the need to make assumptions that introduce observer bias. Given short-term environmental changes, adequately sustaining basic metabolic functions with minimal resource investment may be a more-likely cellular objective on that time scale than, for example, maximizing growth. We therefore did not set an objective function for flux sampling but used four experimentally measured flux values (CO_2_ input and fumarate, malate and starch accumulation) to constrain a leaf metabolic model.^28^ We further demonstrated how these flux sampling results can lead to different conclusion than traditional FBA analyses.

By constraining the model to both cold and control conditions we were able to select reactions that show different flux distributions across the two conditions. Our model highlights reactions that are essential to change with temperature (i.e., the flux distribution of the two temperature conditions do not overlap) such as the production of cytosolic fumarate via malate.^24^ The model further demonstrates the properties of the flux distributions of GABA to differ in cold and control conditions, highlighting GABA as a plausible signaling molecule for supporting a shift in the nitrogen and carbon balance, required to sustain photosynthesis in the cold. Thus, through flux sampling, we were able to generate novel hypotheses about the roles of GABA, fumarate and malate in cold acclimation, which would have been unfeasible to detect using FBA and FVA methods.

By overlaying different FBA solutions onto flux sampling distributions obtained under condition-specific model constraints, FBA in combination with flux sampling, could, in future work, be used to determine plausible objective functions and help generate predictions about how cellular objectives might be changing in response to environmental changes.

## Methods

### COBRA methods for flux sampling

Constraint-based reconstruction and analysis (COBRA) methods for genome-scale metabolic networks are integrated in the COBRA toolbox^53,54^ for the MATLAB programming language and the COBRApy package^55^ for the open-source Python programming language. Three algorithms for flux sampling exist across the two platforms: CHRR (MATLAB), ACHR (MATLAB and Python), and OPTGP (Python). Further flux-sampling algorithms exist;^37,56–58^ however, as they are not currently available in the COBRA packages, they are here not considered for comparison.^59,60^

1. *The artificially centered hit-and-run (ACHR)* sampler estimates the center of the solution space in a “warm-up” phase. This estimate is then continuously revised with further sampling. The center estimate is used to inform the direction of further sampling such that the full solution space is covered in fewer steps than in traditional hit-and-run sampling (where the direction of the next sample is chosen at random).^34^ Although the Markovian nature of hit-and-run (i.e., the fact that each future sampling state is dependent only on the current sampling state) is lost in the ACHR, it overcomes the edge-trapping limitation of the standard hit-and-run algorithm (i.e., it no longer gets stuck at the bounds of a solution space if these are of an elongated shape, a frequent feature in metabolic models).
2. *The optimized general parallel sampler (OPTGP)* is argued to be an improvement on the ACHR, because from the warm-up point it generates multiple short chains from the estimated center and only considers the last point in the chain as a sample.^35^ It thereby increases the randomness and efficiency with which the total solution space is explored. Furthermore, it allows for parallel sampling. Larger samples can thus be generated in shorter run-times.
3. *The coordinate hit-and-run with rounding (CHRR)* algorithm starts with a pre-processing step that rounds the solution space to a more regular, convex shape, and therefore a Markov chain can be used to explore the rounded solution space without the limitation of edge-trapping. After sampling, the solutions are back-transformed to match the original solution space in order to obtain the true value of the sampled points.^33,61^

### Assessing convergence

The aim of flux sampling is to generate enough consecutive samples (a long enough chain) in order to get an accurate depiction of the solution space. A sample chain is considered to have converged once it can be assumed that the sampled subset of solutions represent the properties of the true solutions obtained from an infinite amount of samples (i.e., when the shape of the flux distribution no longer changes with more samples).^62^ In flux sampling, an algorithm’s efficiency is defined by the run-time and the number of samples required for apparent convergence to the true flux distribution. Here, we apply three different diagnostics in order to test for convergence:

1. *Raftery & Lewis diagnostic:* this diagnostic estimates the total number of samples, *N*_max_, required for a set of samples to achieve convergence, based on a given subset of samples (pilot chain).^63^ We estimated *N*_max_ required for convergence using the CODA package in R.^64^ The diagnostic further returns a dependence factor, *I*, which is indicative of autocorrelation (the degree of dependence between consecutive samples). Chains with *I* > 5 are here considered to be problematic as this value suggests high dependence between samples or influential starting values (i.e., the chain was not run long enough).
2. *Interval-based potential scale reduction factor (IPSRF):* adapted from the original Gelman-Rubin diagnostic, PSRF,^40^ the IPSRF is based on comparing the differences between consecutive samples (within sequence interval length) with the total differences observed between all samples (between sequence interval length). Because interval length, rather than variance of the samples (on which the original version is based), is considered, a normal distribution of the samples is no longer a requirement.^42^ As with PSRF, the IPSRF approaches 1 with chain convergence. Here, we consider chains to have failed this convergence diagnostic if IPSRF < 0.9 or IPSRF > 1.1 as calculated using the MCMC Diagnostics toolbox in MATLAB.
3. *Gweke diagnostic:* this diagnostic, as implemented in the R CODA package,^64^ tests whether the mean of the first samples of the chain (10%) is significantly different from the mean of the last set of samples (50%) of the chain.^65^ Assuming that the last set of samples has converged to the stationary distribution, if the two subsets are not significantly different, the entire chain can be considered to have converged. Here we consider chains to have failed this convergence diagnostic if *z* > 1.28.

Because each of the convergence diagnostics tests for different criteria, we use all of the above in order to assure sample convergence. Here, we consider the flux sampling distribution of a reaction to have converged if it passes all of the above criteria.

### COBRA toolbox and COBRApy setup

COBRA Toolbox version 3.0.0 and COBRApy version 0.10.1 were installed on MATLAB R2017a and Python version 3.5.2, respectively.^55^ The linear programming solver used was Gurobi, version 8.0.0. The samplers were used in accordance with COBRA documentation using default parameter settings unless otherwise specified. Although an ACHR implementation is available both in the COBRA toolbox and in COBRApy, the comparisons made here are based on the Python implementation of the ACHR sampler because it is open-source and because the OPTGP, which is available only in Python, directly builds on the general parallel sampler (GP) of the ACHR. We did collect preliminary convergence and run-time data of the ACHR algorithm in MATLAB; however, because its convergence was evidently slower than CHRR, we have chosen to omit its MATLAB implementation from further analyses. OPTGP and CHRR algorithms were run using two parallel processes; there is currently no option to run ACHR as multiple processes.

### Metabolic models

To compare the performance of the samplers, three published genome-scale *A*. *thaliana* metabolic models were obtained in SBML format and are from here on referred to by their first author: the Poolman,^32^ Arnold,^28^ and Dal’Molin^2^ models. The Poolman model is based on the Aracyc database^66^ and describes a non-compartmentalized heterotrophic culture using 1406 reactions. The Arnold model describes a compartmentalized, photoautotrophic system and is based on 549 manually curated *A*. *thaliana* specific reactions. The Dal’Molin model is a compartmentalized network reconstruction of 1601 reactions, applicable to both photosynthetic and non-photosynthetic tissues of plant metabolism.^2^ The original model constraints^2,28,32^ were used when comparing sampler performance across these models, such that the Arnold, Poolman, and Dal’Molin model had 270, 645, and 330 degrees of freedom respectively.

Samples that are close together within a sample chain can be autocorrelated (i.e., be similar to one another due to the way in which the algorithm works). In order to avoid the effects of autocorrelation and to ensure convergence, a large number of samples should be run and a technique called thinning can be applied.^67^ Sample chains that are thinned store only every *k*^th^ sample in the chain, where *T* = *k* is called the thinning constant. In order to compare autocorrelation of the three algorithms, we applied three different thinning constants (*T* = 100, 1000, 10,000) storing 5000 samples for each chain. Three replicate chains were run for each value of *T*. Convergence diagnostics were calculated for sample chains produced for each of the model reactions. Run-times were obtained on a personal laptop (7.2 GB RAM, i5-6200U CPU processor, 4 cores, 2.3 GHz capacity, Ubuntu 16.04.4 OS).

### Experimental data

Carbon assimilation and respiration (CO_2_ influx and outflux) by the *A*. *thaliana* wild-type Columbia-0 (Col-0) was measured using infrared gas analysis at 100 μmol m^−2^ s^−1^ light under control conditions (20 °C) and after 1 week of cold treatment (4 °C) as described previously.^24^ Fumarate, malate and starch concentrations at the onset and at the end of the photoperiod were measured for both control and cold conditions using enzyme assays.^24^ Averages of 3–4 replicated were calculated. Measurements taken across the different conditions were tested for significant differences using an unpaired *t* test (*p* < 0.05) assuming unequal variances, as implemented in R.

Given that previously published data confirm an approximately constant rate of accumulation of transient carbon storage products during the day,^21,24^ we subtracted the beginning of day metabolite concentrations from the end of day concentrations for each of the products in order to obtain a flux value for carbon storage over one photoperiod. These flux values were used to constrain the Arnold model (Fig. 1). This reduced the degrees of freedom of the model to 266.

### Model constraints

Given that the Arnold model is leaf specific and manually curated, it suits the purpose of studying photosynthetic plant acclimation. In order to do so we constrained the model with our experimental diurnal flux data for malate, fumarate, and starch accumulation as well as CO_2_ influx. We set the cytosolic fumarase reaction, which produces cytosolic fumarate from malate, to be reversible.^24^ Outgoing fumarate, malate, and starch reactions were added to the model in order to simulate diurnal carbon storage (Fig. 1). Diurnal accumulation of the metabolites was calculated by subtracting average beginning of day concentrations from average end of day concentration values. Metabolite accumulation and CO_2_ influx were converted to mmol (gFW)^−1^ Day^−1^. The resulting values were applied as model constraints. Upper and lower bounds were applied according to the calculated standard errors of three to four replicates as shown in Fig. 3. To ensure convergence of all flux sampling distributions, 100,000 flux samples with a thinning of 10,000 were generated using the CHRR algorithm in the COBRA Toolbox. A Kruskal–Wallis test, as implemented in the SciPy Python package, version 0.19.1, was used to assess whether flux samples generated using either the cold or the control constrained model stemmed from the same distribution.^68^

### Reporting summary

Further information on research design is available in the Nature Research Reporting Summary linked to this article.

## Supplementary information

reporting summary

## Data Availability

The authors declare that all data supporting the findings of this study are available within the paper and online or can be generated using the publicly available source code (https://github.com/HAHerrmann/FluxSamplingPlantModels; 10.5281/zenodo.3239075).

## Supplementary information

**Supplementary information** accompanies the paper on the *npj Systems Biology and Applications* website (10.1038/s41540-019-0109-0).

## References

[1] Fondi M, Lio P (2015). Multi-omics and metabolic modelling pipelines: challenges and tools for systems microbiology. Microbiol. Res..

[2] Dal’Molin GO, Queck L-E, Palfreyman RW, Brumbley SM, Nielsen LK (2010). AraGEM, a genome-scale reconstruction of the primary metabolic network in Arabidopsis. Plant Physiol..

[3] Boardbar A, Monk JM, King ZA, Palsson BO (2014). Constraint-based models predict metabolic and associated cellular functions. Nat. Rev. Genet..

[4] Orth JD, Thiele I, Palsson BO (2010). What is flux balance analysis?. Nat. Biotechnol..

[5] Feist AM, Palsson BO (2010). The biomass objective function. Curr. Opin. Microbiol..

[6] Yuan H, Cheung M, Hilbers PAJ, van Riel NAW (2016). Flux balance analysis of plant metabolism: the effect of biomass composition and model structure on model predictions. Front. Plant Sci..

[7] Antoniewicz MR (2015). Methods and advances in metabolic flux analysis: a mini-review. J. Ind. Microbiol. Biotechnol..

[8] Budinich M, Bourdon J, Larhlimi A, Eveillard D (2017). A multi-objective constraint-based approach for modeling genome-scale microbial ecosystems. PLoS ONE.

[9] García Sánchez CE, Torres Sáez RG (2014). Comparison and analysis of objective functions in flux balance analysis. Biotechnol. Prog..

[10] Varma A, Palsson BO (1994). Stoichiometric flux balance models quantitatively predict growth and metabolic by-product secretion in wild-type Escherichia coli W3110. Appl. Environ. Microbiol..

[11] Boyle NR, Sengupta N, Morgan JA (2017). Metabolic flux analysis of heterotrophic growth in Chalmydomonas reinhardtii. PLoS ONE.

[12] Fischer E, Sauer U (2005). Large-scale in vivo flux analysis shows rigidity and suboptimal performance of Bacillus subtilis metabolism. Nat. Genet..

[13] Kitano H (2004). Biological robustness. Nat. Rev. Genet..

[14] Kaneko K (2012). Phenotypic plasticity and robustness: evolutionary stability theory, gene expression dynamics model, and laboratory experiments. Adv. Exp. Med. Biol..

[15] Long SP, Ainsworth EA, Leakey AD, Nösberger J, Ort DR (2006). Food for thought: lower-than-expected crop yield stimulation with rising CO_2_ concentrations. Science.

[16] Lobell DB, Cassman KG, Field CB (2009). Crop yield gaps: their importance, magnitudes, and causes. Ann. Rev. Environ. Res..

[17] Schellenberger J, Palsson BO (2009). Use of randomized sampling for analysis of metabolic networks. J. Biol. Chem..

[18] Wiback SJ, Famili I, Greenberg HJ, Palsson BO (2004). Monte Carlo sampling can be used to determine the size and shape of the steady-state flux space. J. Theor. Biol..

[19] Maarleveld TR, Wortel MT, Olivier BG, Teusink B, Bruggeman FJ (2015). Interplay between constraints, objectives, and optimality for genome-scale stoichiometric models. PLoS Comput. Biol..

[20] Brooks SP, Roberts GO (1998). Convergence assessment techniques for Markov chain Monte Carlo. Stat. Comp..

[21] Smith A, Stitt M (2007). Coordination of carbon supply and plant growth. Plant Cell Environ..

[22] Pracharoenwattana I (2010). Arabidopsis has a cytosolic fumarase required for the massive allocation of photosynthate into fumaric acid and for rapid plant growth on high nitrogen. Plant J..

[23] Dyson BC (2015). Acclimation of metabolism to light in Arabidopsis thaliana: the glucose 6-phosphate/phosphate translocator GPT2 directs metabolic acclimation. Plant Cell Environ..

[24] Dyson BC (2016). FUM2, a cytosolic fumarase, is essential for acclimation to low temperature in Arabidopsis thaliana. Plant Physiol..

[25] Küstner L., Nägele T. & Heyer A. G. Mathematical modeling of diurnal patterns of carbon allocation to shoot and root in Arabidopsis thaliana. *Nat*. *Sys*. *Biol*. *Appl*. **5** (2019). 10.1038/s41540-018-0080-1 PMC6346032 30701083

[26] Chia DW, Yoder TJ, Reiter W-D, Gibson SI (2000). Fumaric acid: an overlooked form of fixed carbon in Arabidopsis. Planta.

[27] Zell MB (2010). Analysis of Arabidopsis with highly reduced levels of malate and fumarate sheds light on the role of these organic acids as storage molecules. Plant Physiol..

[28] Arnold A, Nikoloski Z (2014). Bottom-up reconstruction of Arabidopsis and its application to determining the metabolic costs of enzyme production. Plant Physiol..

[29] Dal’Molin CGO, Queck LE, Saa PA, Nielsen LK (2015). A multi-tissue genome-scale metabolic modeling framework for the analysis of whole plant systems. Front. Plant. Sci..

[30] Cheung CYM, Poolman MG, Fell DA, Ratcliffe RG, Sweetlove LJ (2014). A diel flux balance model captures interactions between light and dark metabolism during day-night cycles in C3 and crassulacean acid metabolism leaves. Plant Physiol..

[31] Mintz-Oron S (2012). Reconstruction of Arabidopsis metabolic network models accounting for subcellular compartmentalization and tissue-specificity. Proc. Natl. Acad. Sci. USA.

[32] Poolman MG, Miguet L, Sweetlove LJ, Fell DA (2009). A genome-scale metabolic model of Arabidopsis and some of its properties. Plant Physiol..

[33] Haraldsdottir HS, Cousins B, Thiele I, Fleming RMT, Vempala S (2017). CHRR: coordinate hit-and-run with rounding for uniform sampling of constraint-based models. Bioinformatics.

[34] Kaufman DE, Smith RL (1998). Direction choice for accelerated convergence in hit-and-run sampling. Oper. Res..

[35] Megchelenbrink W, Huynen M, Marchiori E (2014). optGpSampler: an improved tool for uniformly sampling the solution-space of genome-scale metabolic networks. PLoS ONE.

[36] Price ND, Schellenberger J, Palsson BO (2004). Uniform sampling of steady-state flux spaces: means to design experiments and to interpret enzymopathies. Biophys. J..

[37] Bordel S, Agren R, Nielsen J (2010). Sampling the solution space in genome-scale metabolic networks reveals transcriptional regulation in key enzymes. PLoS Comput. Biol..

[38] Mo ML, Palsson BO, Herrgård MJ (2009). Connecting extracellular metabolomic measurements to intracellular flux states in yeast. BMC Syst. Biol..

[39] Shlomi T, Benyamini T, Gottlieb E, Sharan R, Ruppin E (2011). Genome-scale metabolic modeling elucidates the role of proliferative adaptation in causing the Warburg effect. PLoS Comput. Biol..

[40] Gelman, A. et al. Bayesian data analysis, 3rd edn (London, UK: Chapman and Hall/CRC, 2013).

[41] Cowles MK, Carlin BP (1996). Markov chain monte carlo convergence diagnostics: sa comparative review. J. Am. Stat. Assoc..

[42] Brooks SP, Gelman A (1996). General methods for monitoring convergence of iterative simulations. J. Comp. Graph. Stat..

[43] Lundmark M, Cavaco AM, Trevanion S, Hurry V (2006). Carbon partitioning and export in transgenic Arabidopsis thaliana with altered capacity for sucrose synthesis grown at low temperature: a role for metabolite transporters. Plant Cell. Environ..

[44] Strand A, Foyer CH, Gustafsson P, Gardeström P, Hurry V (2003). Altering flux through the sucrose biosynthesis pathway in transgenic Arabidopsis thaliana modifies photosynthetic acclimation at low temperatures and the development of freezing tolerance. Plant Cell Environ..

[45] Strand A (1999). Acclimation of Arabidopsis leaves developing at low temperatures. Increasing cytoplamic volumes accompanies increased activities of enzymes in the Calvin cycle and in the sucrose-biosynthesis pathway. Plant Physiol..

[46] Nägele T, Heyer AG (2013). Approximating subcellular organisation of carbohydrate metabolism during cold acclimation in different natural accessions of Arabidopsis thaliana. New Phytol..

[47] Mazzucotelli E, Tartari A, Cattivelli L, Forlani G (2006). Metabolism of γ-aminobutyric acid during cold acclimation and freezing and its relationship to frost tolerance in barley and wheat. J. Exp. Bot..

[48] Beuve N (2004). Putative role of *γ*-aminobutyric acid (GABA) as a long-distance signal in up-regulation of nitrate uptake in Brassica napus L. Plant Cell Environ..

[49] Michaeli S, Fromm H (2015). Closing the loop on the GABA shunt in plants: are GABA metabolism and signaling entwined?. Front. Plant Sci..

[50] Barbosa JM, Singh NK, Cherry JH, Locy RD (2010). Nitrate uptake and utilization is modulated by exogenous *γ*-aminobutyric acid in Arabidopsis thaliana seedlings. Plant Physiol. Biochem..

[51] Atkinson LJ, Sherlock DJ, Atkin OK (2015). Source of nitrogen associated with recovery of relative growth rate in Arabidopsis thaliana acclimated to sustained cold treatment. Plant Cell Environ..

[52] Müller C, Scheible W-R, Stitt M, Krapp A (2001). Influence of malate and 2-oxoglutarate on the NIA transcript level and nitrate reductase activity in tobacco leaves. Plant Cell Environ..

[53] Schellenberger J (2011). Quantitative prediction of cellular metabolism with constraint-based models: the COBRA Toolbox v2.0. Nat. Protoc..

[54] Heirendt L (2019). Creation and analysis of biochemical constraint-based models using the COBRA Toolbox v.3.0. Nat. Protoc..

[55] Ebrahim A, Lerman JA, Palsson BO, Hyduke DR (2013). COBRApy: COnstraints-based reconstruction and analysis for python. BMC Syst. Biol..

[56] Saa PA, Nielsen LK (2016). ll-ACHRB: a scalable algorithm for sampling the feasible solution space of metabolic networks. Bioinformatics.

[57] Becker NB, Allen RJ, ten Wolde PR (2012). Non-stationary forward flux sampling. J. Chem. Phys..

[58] Damiani C (2014). An ensemble evolutionary constraint-based approach to understand the emergence of metabolic phenotypes. Nat. Comput..

[59] Agren R (2013). The RAVEN toolbox and its use for generating a genome-scale metabolic model for Penicillium chrysogenum. PLoS Comput. Biol..

[60] Damiani C (2017). A metabolic core model elucidates how enhanced utilization of glucose and glutamine, with enhanced glutamine-dependent lactate production, promotes cancer cell growth: the WarburQ effect. PLoS Comput. Biol..

[61] De Martino D, Mori M, Parisi V (2015). Uniform sampling of steady states in metabolic networks: heterogeneous scales and rounding. PLoS One.

[62] Hamra G, MacLehose R, Richardson D (2013). Markov chain Monte Carlo: an introduction for epidemiologists. Int. J. Epidemiol..

[63] Raftery A. E. & Lewis S. M. *“How many iterations in the Gibbs sampler?“* Bernardo J. M., Berger J., Dawid A. P., Smith A. F. M. 4th edn, (Oxford: Bayesian Statistics 1992).

[64] Plummer M, Best N, Cowles K, Vines K (2006). CODA: convergence diagnosis and output analysis for MCMC. R. News.

[65] Gweke J. Evaluating the accuracy of sampling-based approaches to calculating posterior moments. Oxford: J. O. Berger, A. P. Dawid, Smith A. F. M. (ed. 4) Bayesian Statistics: (Clarendon Press 1991).

[66] Mueller LA, Zhang P, Rhee SY (2003). AraCyc: a biochemical pathway database for arabidopsis. Plant Physiol..

[67] Ray J., Pincar A. & Seshadhri C. Are We There Yet? When to Stop a Markov Chain while Generating Random Graphs. International Workshop on Algorithms and Models for the Web-Graph, WAW: Algorithms and Models for the Web Graph, pp 153–164 (2012).

[68] Kruskal WH, Wallis WA (1952). Use of ranks in one-criterion variance analysis. J. Am. Stat. Assoc..

